# Unveiling the Aftermath: Exploring Residue Profiles of Insecticides, Herbicides, and Fungicides in Rice Straw, Soils, and Air Post-Mixed Pesticide-Contaminated Biomass Burning

**DOI:** 10.3390/toxics12010086

**Published:** 2024-01-18

**Authors:** Suteekan Lamnoi, Thirasant Boonupara, Sulak Sumitsawan, Patipat Vongruang, Tippawan Prapamontol, Patchimaporn Udomkun, Puangrat Kajitvichyanukul

**Affiliations:** 1 Department of Environmental Engineering, Faculty of Engineering, Chiang Mai University, Chiang Mai 50200, Thailand; suteekan.lamnoi@gmail.com (S.L.); jarmore001@gmail.com (T.B.); or sulak.sumit@cmu.ac.th (S.S.); 2 Environmental Health, School of Public Health, University of Phayao, Phayao 56000, Thailand; patipat7@hotmail.com; 3 Environmental and Health Research Group, Research Institute for Health Sciences, Chiang Mai University, Chiang Mai 50200, Thailand; tippwawan.prapamontol@cmu.ac.th; 4 Office of Research Administration, Chiang Mai University, Chiang Mai 50200, Thailand

**Keywords:** pesticide residue, agricultural burning, environmental contamination, toxic air pollutants, particulate matter, soil and air pollution

## Abstract

This study delved into the impact of open biomass burning on the distribution of pesticide and polycyclic aromatic hydrocarbon (PAH) residues across soil, rice straw, total suspended particulates (TSP), particulate matter with aerodynamic diameter ≤ 10 µm (PM_10_), and aerosols. A combination of herbicides atrazine (ATZ) and diuron (DIU), fungicide carbendazim (CBD), and insecticide chlorpyriphos (CPF) was applied to biomass before burning. Post-burning, the primary soil pesticide shifted from propyzamide (67.6%) to chlorpyriphos (94.8%). Raw straw biomass retained residues from all pesticide groups, with chlorpyriphos notably dominating (79.7%). Ash residue analysis unveiled significant alterations, with elevated concentrations of chlorpyriphos and terbuthylazine, alongside the emergence of atrazine-desethyl and triadimenol. Pre-burning TSP analysis identified 15 pesticides, with linuron as the primary compound (51.8%). Post-burning, all 21 pesticides were detected, showing significant increases in metobromuron, atrazine-desethyl, and cyanazine concentrations. PM_10_ composition mirrored TSP but exhibited additional compounds and heightened concentrations, particularly for atrazine, linuron, and cyanazine. Aerosol analysis post-burning indicated a substantial 39.2-fold increase in atrazine concentration, accompanied by the presence of sebuthylazine, formothion, and propyzamide. Carcinogenic PAHs exhibited noteworthy post-burning increases, contributing around 90.1 and 86.9% of all detected PAHs in TSP and PM_10_, respectively. These insights advance understanding of pesticide dynamics in burning processes, crucial for implementing sustainable agricultural practices and safeguarding environmental and human health.

## 1. Introduction

Pesticides, which include insecticides, bactericides, and herbicides, are categorized based on their distinct functions and chemical structures such as organo-phosphorus, organo-chlorines, nitrogen-benzenes, phenols, metallo-organics, and other compounds. Playing a vital role in modern agriculture to meet global food demand, pesticides are crucial for eliminating undesirable organisms. Pesticide usage rates vary across the globe, even within the same region. Asia, in particular, has the highest observed average rate of pesticide use [1]. Despite their significance, a mere fraction, approximately 1%, of applied pesticides reaches the intended target pest, with the majority seeping into soil, water, and air. This pervasive environmental infiltration poses substantial risks to ecosystems, biodiversity, and human health [2,3]. Recognized for their potential to act as mutagens, pesticides pose a significant threat to human health due to their constituents being capable of triggering DNA deviations. According to the World Health Organization (WHO), approximately 1,000,000 people unintentionally suffer from acute pesticide poisoning annually, particularly in low- and middle-income countries, leading to a death rate ranging from 0.4% to 1.9% [4,5,6,7,8]. Work-related exposure to pesticides is implicated in 70% of these fatalities. Furthermore, prolonged exposure to lower pesticide doses is associated with various long-term health issues, including tumors and nervous system disorders [9,10].

Soil, a vital component in the ecosystem, intricately influences the fate, behavior, and dispersion of chemical pesticides [11]. As the primary repository for pesticides used in agriculture, soil serves as a sponge, absorbing most pesticides and their degradation products, consequently impacting various food webs. The runoff of pesticides from soils into water sources and their volatilization into the atmosphere present significant environmental challenges, adversely affecting both air and surface water quality [10,12]. Volatilization, involving the transformation of pesticides from a liquid or solid state to a gaseous state, carries implications for air quality, allowing these volatile substances to travel and contaminate over long distances [4,13]. Furthermore, runoff and flooding also contribute to the unintentional diffusion and non-target contamination of pesticides [14,15]. Globally, varying levels of pesticides, as documented by Cech et al. [16], Panis et al. [17], Peris et al. [18], Sun et al. [19], and Vickneswaran et al. [20], pose threats to both human health and the environment.

Open burning, defined as the direct emission of combustion products into the ambient air without proper containment [21], encompasses various practices such as burning crop residues, using firewood in cooking stoves, and incinerating domestic and industrial wastes. Asia, particularly, has been a major contributor, accounting for almost 50% of total biomass burned, with rice, corn, and sugarcane burning being prominent [22,23,24]. The consequences of crop residue burning extend beyond the immediate release of pollutants like sulfur dioxide (SO_2_), carbon monoxide (CO), nitrogen oxides (NO_x_), and polycyclic aromatic hydrocarbons (PAHs) into the atmosphere, affecting air quality and posing health risks [24,25,26]. In Thailand, Pothirat et al. [27] associated increased particle concentrations during the dry season in the northern region with elevated mortality rates. While understanding the long-term health effects remains a complex challenge, biomass burning’s contribution to particulate matter (PM) is known to induce premature death, especially in individuals with heart or lung diseases, causing nonfatal heart attacks, irregular heartbeats, aggravated asthma, decreased lung function, and increased respiratory symptoms [28,29]. Adding to these concerns, Kongpran et al. [30] investigated the impact of PAHs on human health in the northern part of Thailand. Their study, while not explicitly linking PAHs to biomass burning, noted that PAHs could occur during the seasonal haze episodes associated with combustion-related smoke emissions. Furthermore, certain metals and trace elements, which exhibit a notable surge during episodes of elevated PM levels from biomass burning, could potentially pose additional health risks to the human population.

The existing research landscape lacks a thorough exploration of the environmental consequences associated with open burning of agricultural biomass contaminated with a variety of pesticides. Most current studies tend to concentrate either on investigating pesticide residues post-application in the field or on evaluating the combustion characteristics of biomass residues during open burning. For instance, Junpen et al. [31] delved into the levels of air pollutant emissions resulting from open-space rice straw burning, while Tipayarom and Oanh [32] examined the impact of rice straw open burning on the levels and profiles of semi-volatile organic compounds in ambient air. Akbari et al. [33] focused on assessing the emission factors of ash and metals bound with PM_2.5_ released from burning various biomass types in a constructed open-system combustion chamber. Additionally, Chen et al. [34] provided a review summarizing and analyzing techniques used to determine toxic products released during the thermal decomposition of pesticides. Meanwhile, a study by Růžičková et al. [35] analyzed the occurrence of pesticides and their residues in char produced by the combustion of wood pellets in domestic boilers. Despite these efforts, none of these studies have thoroughly explored the potential contributions to soil, ash, and atmospheric pollution during the open burning of pesticide-laden agricultural residues. Addressing this research gap is crucial for understanding the complex interplay between different pesticides, combustion processes, and resulting contamination levels in diverse environmental matrices. Therefore, the objectives of this study were to assess the extent of pollution caused by open burning of mixed pesticide-contaminated rice straw biomass, investigate the distribution and contamination of pesticides in soil and ash residues, and analyze the associated atmospheric impact. The emphasis on the specific scenario was particularly notable, especially in situations where farmers might neglect withdrawal periods by applying pesticides just a few days before post-harvesting. Through a systematic investigation of these objectives, the aim was to contribute to a more comprehensive understanding of the environmental and possible health implications associated with open burning practices involving pesticide-laden agricultural residues.

## 2. Materials and Methods

### 2.1. Raw Materials and Chemicals

In this investigation, rice straw was selected as the primary biomass material for open burn testing, originating from a local farm in Sanpatong district, Chiang Mai, Thailand. The pesticides atrazine, diuron, carbendazim, and chlorpyrifos were employed in the experiments, sourced from reputable local suppliers: ICP Ladda Co., Ltd. in Bangkok, Thailand; Khowtongseang Co., Ltd. in Pichit, Thailand; Millennium Farm Co., Ltd. in Nakhonpathom, Thailand; and K.T.S. Power Co., Ltd. in Rayong, Thailand, respectively. The detailed main chemical composition of all pesticides can be found in Table 1. Nevertheless, it is crucial to highlight that several other compounds were also identified in the mixed pesticides, albeit at very low concentrations. These included triadimefon, triadimenol, prochloraz, chlorotoluron, ametryn, and so forth.

All solvents required for extraction and analysis were procured from RCI Labscan in Bangkok, Thailand. For purification purposes, the QuEChERS Extraction Kit, comprising magnesium sulfate, sodium chloride, sodium citrate, and disodium citrate sesquihydrate, along with 2 mL of QuEChERS dispersive solid-phase extraction (SPE) featuring primary secondary amine (PSA), octadecylsilane end-capped, and magnesium sulfate, were employed. These materials were sourced from Agilent, based in Agilent Technologies, Santa Clara, CA, USA. The specified herbicides (atrazine-desethyl, cyanazine, simazine, atrazine, propazine, sebuthylazine, deisopropylatrazine, terbuthylazine, 2,4-dichlorophenoxyacetic acid (2,4-D), diuron, linuron, metobromuron, and ametryn), fungicides (carbendazim, triadimefon, kresoxim methyl, triadimenol, and prochloraz), and insecticide (chlorpyriphos, formothion, and propyzamide) were considered standard references. These pesticides, each with a purity surpassing 99%, were procured from CPAchem Ltd. located in Bogomilovo, Bulgaria.

Furthermore, a mixed solution of 16 EPA-PAHs, comprising naphthalene (Nap), acenaphthylene (Acy), acenaphthene (Ace), fluorene (Flu), phenanthrene (Phe), anthracene (Ant), fluoranthene (Fla), pyrene (Pyr), benzo[a]anthracene (BaA), chrysene (Chr), benzo[b]fluoranthene (BbF), benzo[k]fluoranthene (BkF), benzo[a]pyrene (BaP), indeno[1,2,3,c-d] pyrene (IcdP), dibenz[a,h]anthracene (DahA), and benzo[ghi]perylene (BghiP), was acquired from AccuStandard in Connecticut, USA, at a concentration of 200 mg/L in a dichloromethane solution.

### 2.2. Burning Facility

The burning of rice straw biomass was conducted in a specialized open burn test facility situated in Sanpatong district, Chiang Mai, Thailand, precisely at coordinates 18.6385° N and 98.8367° E. The designated burning area covered 30 × 30 m^2^. Owing to the flat topography of the experimental site, wind flow rates during the burning tests varied between 8.0 and 11.3 km/h, causing fluctuating wind directions. Throughout the burning experiments, the ambient temperature ranged from 27 to 32 °C.

### 2.3. Experimental Burning Procedure

Approximately 20 kg of rice straw biomass, configured with dimensions of 1.0 × 1.0 × 0.5 m^3^ (width, length, and height), underwent meticulous preparation. The biomass was uniformly coated using a hand sprayer and left to air-dry outdoors overnight, ensuring optimal conditions. To maintain consistency and account for potential seasonal variations, the entire test program was confined to a four-day timeframe.

The rice straw biomass underwent pesticide treatment following recommended concentrations for paddy field applications as specified by manufacturers. Atrazine, diuron, carbendazim, and chlorpyriphos were individually prepared at concentrations of 540, 450, 750, and 5000 ppm, respectively, before being combined in equal proportions at a ratio of 1:1:1:1. The group of herbicides, fungicides, and insecticides were combined following the application practices commonly used by farmers in the field. The 20 kg biomass was methodically sprayed with 500 mL of the mixed pesticides. Ignition was initiated at a single point strategically positioned at the center of the base, taking approximately 5 s for the establishment of self-sustained combustion.

**Figure 1:**
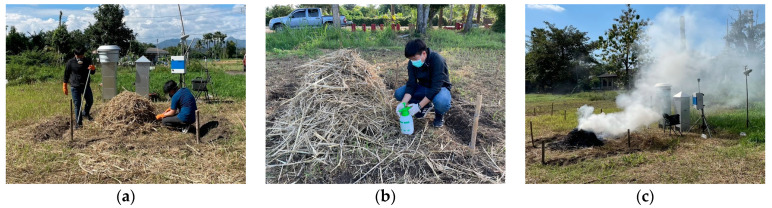
The procedure of open burning process: (**a**) biomass preparation, (**b**) pesticide spraying onto biomass, and (**c**) incomplete combustion and air sampling.

To ensure a significant production of smoke, incomplete combustion was maintained for a nominal flaming burn time of 20 min. Sampling extended beyond this period, reaching up to 5 h until no visible smoldering persisted. To prevent potential cross-contamination, experiments commenced with a minimum separation distance of 8 m between individual test areas. The specific characteristics of the burning process as relevant to this study are displayed in Figure 1. The burning process was performed in triplicate.

### 2.4. Sampling Procedures

#### 2.4.1. Soil and Biomass Residue Sampling

Sampling of topsoil from depths of 0–15 cm was conducted both before and after the open burning test. This involved collecting soil from three randomized positions within each designated area, resulting in composite samples weighing approximately 500 g each. Simultaneously, rice straw samples, around 100 g each, were obtained before combustion, along with ash residues generated after the burning experiments. All collected samples, comprising soil and biomass residues, were carefully placed within amber zip-lock bags. Each bag was meticulously labeled for precise identification, and the samples were swiftly transported to the laboratory. Subsequently, these samples were stored at room temperature for a 7-day equilibration period before undergoing further extraction and analytical procedures.

#### 2.4.2. Air Sampling

In this study, air samples were collected under two conditions: during biomass burning and in the atmosphere without burning. Total suspended particulate (TSP) samples were collected using 20.3 × 25.4 cm glass fiber filters (ADVANTEC, Tokyo, Japan), while PM_10_ was gathered using 20.3 × 25.4 cm quartz fiber filters (ADVANTEC, Tokyo, Japan). Both samples were acquired using a high-volume sampler following the protocols specified in the US EPA Federal Reference method IO-2.1 [36]. To simulate the inhalation route of Thai residents, the sampler was positioned at a height of approximately 1.6 m and located about 1.5 m from the burning area. Sampling occurred over a 30 min duration at an average flow rate of 1.1 m^3^/min. After the sampling process, the filters were carefully retrieved, shielded in aluminum foil for preservation, and sealed in amber zip-lock bags. They were then transported cool to the laboratory and stored in a freezer at −20 °C until the analysis commenced.

Aerosol samples were meticulously collected using low-volume samplers, following the established procedures outlined in the US EPA Federal Reference method TO-10A [37]. Positioned at a height of approximately 1.6 m from the ground, these samplers operated at 30-min intervals, maintaining a flow rate of about 1.1 m^3^/min. To collect both particles and gases simultaneously, a 75 mm-long glass tube (ORBO™ 49P OSHA Versatile Sampler, Merck KGaA, Darmstadt, Germany) with a 13-mm outer diameter at the inlet end tapered to 8 mm at the outlet end was employed, followed by specially cleaned XAD^®^-2 material (Merck KGaA, Darmstadt, Germany) positioned within a tube. After sampling and before the extraction process, the filters and adsorbents were secured in clean amber zip-lock bags and stored in a dark environment at −20 °C. The effectiveness of this cleaning procedure was validated through the use of blank samples.

### 2.5. Extraction Methods

#### 2.5.1. Soil and Biomass Residue Extraction

The extraction process for soil and biomass residues adhered to the Quick, Easy, Cheap, Effective, Rugged, and Safe (QuEChERS) method. About 10 g of soil or 1 g of biomass was precisely weighed and placed in a 50-mL centrifuge tube. Subsequently, 10 mL of water was added, and the mixture stood at ambient temperature for 30 min. Following this, 10 mL of acetonitrile was introduced. The tube, containing a buffer-salt mixture of 4 g magnesium sulfate anhydrous (MgSO_4_), 1 g sodium chloride, and 0.5 g disodium hydrogen citrate sesquihydrate, was sealed, vigorously shaken for 1 min, and then centrifuged (Multi Centrifuge, VARISPIN 4, NOVAPRO Co., Ltd., Seoul, Republic of Korea) at 4000 rpm for 5 min. Subsequently, 1 mL of the acetonitrile phase was transferred into a separate centrifuge tube with 150 mg of MgSO_4_ and 25 mg of primary-secondary amine (PSA). After sealing, shaking vigorously for 30 s, and centrifugation (Microliter and Haematocrit Centrifuge, NF 480, NÜVE SANAYİ MALZEMELERİ İMALAT VE TİCARET A.Ş, Ankara, Turkey) at 4000 rpm for 5 min, the residue was reconstituted with 0.5 mL of acetonitrile. Each final extract was filtered through a 0.2 µm membrane filter into a 2 mL amber glass vial, stored at −20 °C until HPLC-MS/MS analysis.

#### 2.5.2. Particulate and Aerosol Sample Extraction for Pesticides Determination

For TSP and PM_10_, samples were weighed, cut into small pieces, and placed in 50 mL centrifuge tubes. The QuEChERS method facilitated extraction. Aerosol samples from the combined XAD^®^-2 material and filter underwent extraction using a Soxhlet extraction method, following the US EPA Federal Reference method TO-10A [37], with slight modifications. The extraction process involved 100 mL of ethyl acetate as the solvent, with an extraction time of 8 h, pre-determined. After undergoing a specialized Soxhlet extraction (Lab Valley Co., Ltd., Bangkok, Thailand), the resulting solution was adjusted to a final volume of 2 mL before injection into 2 mL chromatography vials, stored at −20 °C before injection into the chromatographic system.

#### 2.5.3. Extraction of Particulate and Aerosol Samples for PAH Determination

Each sample had three pieces of 20 mm filters from TSP and PM_10_ collections placed into a 20 mL glass vial. Adding a mixed internal standard of PAHs, acenaphthene D10, perylene D12 (Dr. Ehrenstorfer, LGC, Augsburg, Germany), and 10 mL dichloromethane initiated ultrasonic cleaning. The extract was filtered by a PTFE syringe filter (13 mm, 0.2 µm) and concentrated by a Buchi Heating bath B-491/Buchi Rotavapor R-210 (BUCHI Labortechnik AG, Flawil, Switzerland) at 35 °C to reduce the sample volume to approximately 0.5 mL. The extracts were then adjusted in volume to 1 μL with ethyl acetate before GC-MS analysis.

### 2.6. Chromatographic Determination

#### 2.6.1. HPLC-MS/MS Analysis for Pesticides Determination

**Figure 2:**
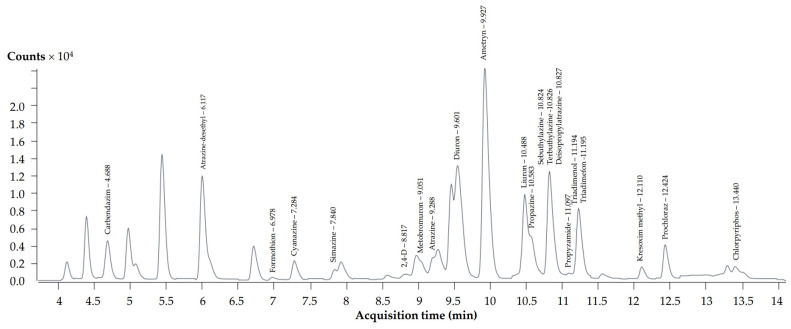
HPLC–MS/MS chromatogram of studied pesticides.

The HPLC–MS/MS system utilized in this analysis included a 1290 vialsampler (G7129B, Agilent, Santa Clara, CA, USA), a 1290 high-speed pump (G7129A, Agilent, CA, USA), and a 1290 MCT detector (G7166B, Agilent, CA, USA). Separation occurred on a Phenomenex Luna C18(2) column (150 mm × 2.00 mm ID, particle size 5 µm). The eluents comprised water with 0.1% formic acid and 5 mM ammonium acetate (A), and methanol with 0.1% formic acid and 5 mM ammonium acetate (B). The flow rate was set at 0.3 mL/min. The gradient conditions were: 0–2.5 min, linear transition from 10% to 40% B; 2.5–12 min, linear transition from 40% to 95% B; 12–13 min, linear transition from 95% to 10% B. Autosampler and column temperatures were 4 and 40 °C, respectively, with a 2 µL injection volume. Detection used Agilent Jet Stream electrospray ionization (ESI) in the positive mode. Operating conditions: capillary voltage 3200–3800 V, nebulizer pressure 45 psi, sheath gas temperature 400 °C, sheath gas flow 12 L/min, gas temperature 300 °C, gas flow 3 L/min. Collision gas pressure and tube lens offset voltages were optimized for each pesticide using the automated optimization procedure in syringe infusion mode. Mass spectrometry scanning used dynamic Multiple Reaction Monitoring (MRM). The method validation included selectivity, linearity, limit of detection (LOD), and limit of quantification (LOQ). The selectivity of the HPLC system was evaluated using standards of the mixed pesticides mentioned. Linearity was determined with duplicate injections of pesticide standards at different concentrations. LODs were estimated per IUPAC Harmonized Guidelines [38], with calculated LODs ranging from 0.00 to 1.24 ng/mL (Table 2) and LOQs from 0.00 to 22.83 ng/mL for all pesticides. Validation indicated significant linear regression (*p* < 0.05) with high determination coefficient values (R^2^ ≥ 0.998), demonstrating reliability. Figure 2 presents a chromatogram illustrating the presence of pesticides. The analysis was performed in triplicate.

#### 2.6.2. GC-MS Analysis for PAH Determination

**Figure 3:**
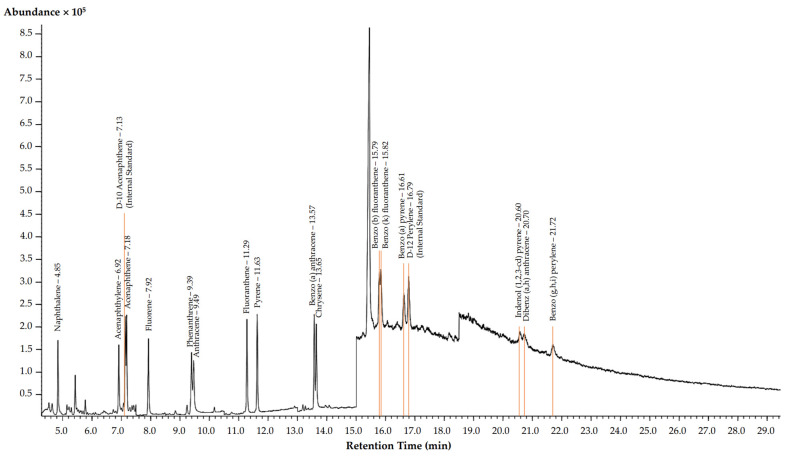
GC-MS chromatogram of studied PAHs.

For PAH contamination in TSP and PM_10_ samples, a gas chromatography-mass spectrometry (GC-MS) using an Agilent 7890A gas chromatograph (Agilent, CA, USA) was conducted. A capillary column (30 m × 0.25 mm × 0.25 μm; Agilent, CA, USA) used helium as the carrier gas at 1.0 mL/min. Injection volume was 1 μL in spitless mode, with injector temperature at 275 °C. The oven temperature program started at 70 °C for 0.5 min, increased at 20 °C/min to 150 °C, then 10 °C/min to 285 °C, and held at 310 °C for 6 min, completing the 27.75-min analysis. GC-MS used electron impact ionization at 70 eV, with the ion source at 250 °C. The selected reaction monitoring (SRM) mode confirmed compound identity. Figure 3 exhibits a chromatogram detailing the presence of PAHs. PAH concentrations were determined with a 7-point calibration curve using deuterated PAHs as internal standards, expressed in ng/m^3^. The LODs and LOQs for each analyte were determined, ranging from 0.54 to 2.65 ng/mL and 1.05 to 4.72 ng/mL, respectively. PAH-spiked sample analysis showed recoveries of 46% to 160%. Validation results confirmed significant linear regression (*p* < 0.05) with strong determination coefficient values (R^2^ ≥ 0.998), affirming the accuracy and reliability of this analytical approach for determining PAH concentrations in air samples. The analysis was conducted in triplicate.

## 3. Results

### 3.1. Variations in Soil Pesticide Levels before and after Rice Straw Burning

The concentration and diversity of pesticides in the soil and straw biomass were systematically examined both before and after the burning process, with comprehensive results detailed in Table 2. In the untreated soil, propyzamide, classified as an insecticide, emerged as the predominant pesticide, constituting 67.6% of the total. It was followed by formothion, also an insecticide, at 19.3%, triadimefon, categorized as a fungicide, at 6.1%, and 2,4-D at 5.6%. Following the controlled burning of biomass-contaminated soil, notable alterations in pesticide concentrations were observed. Specifically, cyanazine, propyzamide, and triadimefon experienced significant reductions of 9.5, 10.3, and 2.8 times, respectively. Notably, herbicide 2,4-D and insecticide formothion became undetectable in the post-burning analysis. Upon closer examination post-burning, additional pesticides spanning various groups were identified in the soil, including chlorpyriphos, atrazine, kresoxim methyl, and atrazine-desethyl. Among these, the insecticide chlorpyriphos emerged as the predominant species, constituting a substantial 94.8% of the total, with the herbicide atrazine following at 4.6%.

### 3.2. Variations in Pesticide Levels in Rice Straw before and after Burning

In the raw straw biomass, residues of all pesticide groups persisted after harvesting rice. The insecticide chlorpyriphos dominated, constituting the highest concentration at 79.7%, followed by terbuthylazine at 15.2% and 2,4-D at 3.9%, respectively. Upon analysis of the ash residue, intriguing alterations in pesticide levels were observed. The concentrations of chlorpyriphos, and terbuthylazine demonstrated increases of 1.5 and 1.6 times, respectively, in comparison to the levels present in the raw biomass. In contrast, herbicide 2,4-D and triadimefon underwent a significant reduction, decreasing by 1.9 and 7.6 times in the ash residue compared to its concentration in the raw biomass. Remarkably, the herbicides cyanazine and atrazine-desethyl and fungicide triadimenol exhibited contrasting fates: cyanazine became untraceable in the biomass after burning, while atrazine-desethyl and triadimenol, previously absent in the raw biomass, was detected in the ash residue.

### 3.3. Variations in Pesticide Levels in Air before and after Rice Straw Burning

Table 3 presents a comprehensive overview of the types and concentrations of detected pesticides in TSP, PM_10_, and aerosol both before and after the burning of biomass contaminated with mixed pesticides. Within the pre-burning TSP, 15 out of the 21 compounds were identified. Herbicide linuron dominated as the primary compound, constituting a significant 51.8% of the total, followed by prochloraz (fungicide) at 15.9%, triadimenol (fungicide) at 12.9%, and atrazine (herbicide) at 7.0%. In the post-burning analysis, 21 investigated compounds were observed in TSP. The concentrations of all pesticides detected in the pre-burning samples showed a substantial increase, except for linuron and kresoxim methyl, which exhibited a noteworthy decrease by 8.9 and 2.1 times, respectively. The change was especially notable in the instances of herbicides metobromuron, atrazine-desethyl, and cyanazine, initially detected at low concentrations in the pre-burning TSP, showing remarkable increases of 479.7, 179.9, and 149.0 times, respectively, in TSP after the burning process. Moreover, certain compounds such as insecticide chlorpyriphos, fungicide carbendazim, and herbicides propazine and terbuthylazine, which were not detected in the pre-burning sample, were discovered in TSP post-burning at significantly elevated concentrations. When considering all detected pesticides, chlorpyriphos stood out as the primary compound, constituting a substantial 24.8% of the total in the analyzed samples. It was closely followed by atrazine at 18.8%, propazine at 9.5%, and carbendazim at 8.5%, each contributing distinctively to the pesticide composition.

Aligning with the pattern observed in TSP, 14 out of the 21 compounds were identified in PM_10_. Of significance, the presence of herbicides deisopropylatrazine and metobromuron in the pre-burning TSP was absent in the pre-burning PM_10_. In the pre-burning PM_10_, only herbicide and fungicide groups were detected. Linuron dominated the composition, constituting 35.7% of the total, followed by atrazine at 30.3%, triadimenol at 16.1%, and prochloraz at 12.0%, each contributing distinctly to the composition. Following the burning of biomass contaminated with mixed pesticides, all 21 compounds were detected in PM_10_. Herbicide atrazine emerged as the dominant compound at 41.2%, followed by linuron at 7.3%, propazine at 7.1%, and insecticide chlorpyriphos at 7.1%. The concentrations of most compounds exhibited significant increments compared to pre-burning samples. For instance, herbicides cyanazine, atrazine-desethyl, and terbuthylazine demonstrated substantial increases of 106.7%, 54.5%, and 38.1%, respectively. However, a reduction of about 2.0 times was observed in linuron and kresoxim methyl. Interestingly, the concentrations of atrazine, linuron, triadimenol, prochloraz, formothion, and propyzamide detected in PM_10_ after burning biomass contaminated with mixed pesticides were higher than those in TSP samples.

Regarding aerosols, atrazine was the only discernible pesticide in the pre-burning phase. However, subsequent to the burning of biomass contaminated with mixed pesticides, a notable change transpired. The concentration of atrazine exhibited a substantial increase, approximately 39.2 times higher than the pre-burning level. Additionally, post-burning analysis revealed the presence of herbicide sebuthylazine and insecticides formothion and propyzamide, collectively constituting around 2.5% of all detected pesticides. While the concentrations of these identified pesticides in aerosols were lower than in TSP and PM10, acknowledging and emphasizing their presence in aerosol samples is crucial. Notably, no fungicide was detected in the aerosol samples.

### 3.4. Changes in PAH Concentrations in TSP and PM_10_ before and after Rice Straw Burning

Table 4 displays the types and concentrations of PAHs detected in TSP and PM_10_ samples. In the pre-burning TSP, 10 out of 15 PAHs were identified, with four of them registering concentrations below the detection limit. Ace dominated the PAH profile at 46.7%, followed by BaA and Ant, contributing 13.4 and 9.4%, respectively. After the burning of biomass contaminated with mixed pesticides, a more complex scenario unfolded. In TSP, thirteen distinct PAHs were identified, with carcinogenic compounds, including BaA, Chr, BbF, BaP, IcdP, and DahA, collectively contributing about 90.1% of all detected PAHs. Following the burning of biomass contaminated with mixed pesticides, there were significant increases in the concentrations of carcinogenic PAHs in TSP, especially BaA and BaP, which were elevated by 108.4 and 137.9 times, respectively. Additionally, carcinogenic PAHs such as IcdP and DahA, absent in the pre-burning TSP, were conspicuously detected in the post-burning samples.

The concentrations of the examined PAHs in PM_10_ samples are presented both before and after the burning of biomass contaminated with mixed pesticides. In the pre-burning PM_10_, twelve out of fifteen types of PAHs were detected, with four of them recognized as carcinogenic compounds. Similar to TSP, Ace (45.7%) was the main dominant in PM_10_, followed by Pyr (12.9%) and Acy (9.9%), respectively. Following the burning of biomass contaminated with mixed pesticides, the concentrations of BbF, Ant, and Chr emerged as the top three dominant PAHs in PM_10_. Interestingly, the concentrations of BbF and BaP increased by approximately 47.6 and 41.3 times, respectively, compared to the pre-burning sample. It is essential to highlight that Chr, DahA, and BghiP, which were undetected in the pre-burning samples, were found in PM_10_ after burning biomass contaminated with mixed pesticides. Furthermore, it is noteworthy that the concentration of PAHs detected in PM_10_ after burning was lower than those in TSP.

## 4. Discussion

While crop residue burning in Asia, particularly in Thailand, provides short-term benefits like improved nutrient uptake and crop yields, it poses lasting threats to soil health, including nutrient depletion, diminished soil biota, and erosion [39]. Uncontrolled fires, whether wildfires or intentional veld fires, can lead to adverse environmental effects over time. If pesticides contaminate the soil or crop residues before burning, especially in cases where farmers may neglect withdrawal periods and apply pesticides just a few days before post-harvesting, the resulting consequences can vary, potentially affecting environmental and human health. The excessive application of pesticides, through methods like spray drift and surface runoff, leads to their accumulation in soil, sediments, and food, infiltrating both ground and surface water [10,40,41]. Contaminated soils harbor hazardous compounds from applied pesticides and their degradation products [42]. This study’s results align, showing a mix of pesticides contaminating raw soil before burning.

### 4.1. Existence of Pesticides in Soil after Burning

Following the combustion process, distinctive trends emerged within the insecticide group. Propyzamide and formothion exhibited either a decrease or became undetectable in the soil post-burning, while chlorpyriphos demonstrated a substantial increase. These findings hint at potential variations in degradation rates, suggesting that propyzamide and formothion are more susceptible to the burning process. In a broader context, the thermochemical conversion of biomass can be theoretically segmented into four stages: drying, pyrolytic decomposition, gas combustion, and char oxidation [43].

The initial preheating of biomass is a critical step that involves the removal of water content within the biomass, typically accomplished at temperatures up to 200 °C. Following this, slow pyrolysis (carbonization) occurs in the temperature range of 300–450 °C, and fast pyrolysis takes place within the range of 500–800 °C. Gasification, conversely, occurs at higher temperatures, typically in the range of 800–1000 °C. Additionally, high-temperature steam gasification (>1000 °C) is followed by combustion in specific processes. The specific temperature conditions and the presence of oxygen play crucial roles in determining the outcomes during the thermal decomposition of biomass [44,45]. Biomass, whether containing chemical substances like pesticides or not, needs exposure to thermal energy to facilitate decomposition into smaller molecules known as volatile compounds. This concept could potentially apply to propyzamide and formothion. These volatile compounds may exist in either solid or gaseous states and are released from the biomass during this process. Following release, these compounds undergo transformations into various products [25], as observed in the case of chlorpyriphos.

The presence of chlorpyriphos residues in the soil highlights its potential for transfer from biomass to soil, persisting even after burning. According to John and Shaike [46], chlorpyriphos exhibits characteristics such as bioaccumulation, high lipophilicity, long-range transport potential, and high persistence, with a strong adsorption to soil and organic matter (K_oc_ > 5000). This is concerning, considering chlorpyrifos is classified as “moderately toxic” (class II) with a half-life in soil ranging from 10–120 days, influenced by various factors [34,47,48]. Extensive evidence suggests that chlorpyrifos may impact multiple human systems due to its high mammalian toxicity [49,50,51,52,53,54]. Additionally, chlorpyrifos can undergo hydrolysis in soil, converting to 3,5,6-trichloro-2-pyridinol (TCP) [48]. Accumulated TCP in soil, known for its antimicrobial property, may hinder microorganisms involved in chlorpyrifos degradation [34,55]. Being a major degradation product, TCP’s higher water solubility than its parent molecule leads to widespread soil and waterborne contamination [56,57,58]. The detection of TCP in human breast milk and foodstuff underscores the urgency of environmental abolition to prevent adverse health effects, as reflected in the bans implemented by 35 countries as of March 2021 [48].

In addition to the insecticide group, a consistent trend emerged within both the fungicide and herbicide groups. This pattern featured the initial presence of atrazine, atrazine-desethyl, and kresoxim methyl, a reduction in the concentration of triadimefon and cyanazine, and the absence of 2,4-D post-burning biomass contaminated with mixed pesticides. The observed variable stability during burning could lead to the thermal decomposition of specific pesticides through incomplete combustion. The emergence of certain pesticides like atrazine and atrazine-desethyl after burning might be attributed to the initial pesticide mixture. However, multiple factors, including burning conditions, combustion byproducts, and chemical transformations, could also contribute to these changes [59]. Despite these complexities, it is clear that pesticide fires in biomass pose ongoing challenges to soil health. Focusing on atrazine, a widely used herbicide, it poses significant concerns for soil integrity due to its extended half-life of approximately 4–57 weeks in soil, and even longer in sediment [60]. This extended persistence categorizes atrazine as a persistent organic pollutant in the environment [61]. The prolonged toxicity and residual effects of atrazine pose a threat to the sustainability of agricultural soils, particularly due to their detrimental impact on soil microbiota [62,63].

### 4.2. Existence of Pesticides in Ash after Burning

Similar to the soil matrix, the ash residue exhibited increased concentrations of specific pesticides, such as chlorpyriphos and terbuthylazine, implying partial degradation during biomass burning. In contrast, 2,4-D and triadimefon levels notably decreased in the ash, indicating susceptibility to thermal decomposition, especially in incomplete combustion conditions. Higher temperatures (>40 °C) were found to accelerate the degradation of 2,4-D, as highlighted by Muhammad et al. [64] and Lawal et al. [65]. The reduction in triadimefon levels may be associated with the emergence of triadimenol, aligning with Singh’s [66] observations of rapid triadimefon degradation at elevated temperatures (>35 °C). The presence of atrazine-desethyl in the ash could be linked to its initial presence in the pesticide mixture applied to biomass or its degradation pathway [67].

Examining the aftermath of open burning in the field, numerous studies have explored how the aging process influences the adsorptivity of ashes produced from the burning of crop residues. Yadav et al. [68] investigated sugarcane trash ash, enhancing atrazine and fipronil degradation in clay loam and loam soils. Similarly, Kumar and Singh [69] explored wheat straw ash and rice straw ash for pretilachlor and sulfosulfuron adsorption, influenced by pH, temperature, and ash surface area. Given that crop residues, whether contaminated or uncontaminated with pesticides, are frequently subjected to burning in agricultural fields, the presence of ashes may significantly immobilize pesticides in the soil [70]. This process may impact pesticides’ environmental fate, raising concerns about their persistence and mobility in agriculture. Prior to immobilization or degradation via ash, pesticide residues in soil and burned biomass might migrate, contaminating offsite locations.

### 4.3. Presence of Pesticides as Air Pollutants

In the realm of air quality, open burning introduces a notable composition of air pollutants, primarily marked by heightened levels of ambient PM, constituting approximately 80–90% of the mass concentration. Additionally, these emissions involve a range of harmful gaseous elements and particulate-phase compounds, such as SO_2_, CO, NO_x_, PAHs, and others. [23,24,71]. While prior investigations have extensively focused on the emission profiles of PM, PAHs, heavy metals, and analogous components stemming from open biomass burning, the presence of pesticides in the aftermath of such burnings has been notably disregarded. This investigation underscores the identification of 21 distinct types of pesticides in both TSP and PM_10_ subsequent to biomass combustion. Significantly, the findings unveil a notable increase in post-burning pesticide concentrations, specifically observed in atrazine-desethyl and cyanazine within TSP and PM_10_. Certain compounds—chlorpyriphos, carbendazim, propazine, and terbuthylazine in TSP, and deisopropylatrazine and metobromuron in PM_10_—previously absent in pre-burning samples were detected after burning. Concerning aerosols, it is crucial to highlight that the detection of compounds such as chlorpyriphos, carbendazim, propazine, and terbuthylazine after biomass burning signals their release into the atmosphere, incorporating them into the aerosol during combustion.

These outcomes not only suggest the release of stored pesticides from the combusted biomass but also indicate potential decomposition, transformations, or the generation of breakdown products during the combustion process. As emphasized by Růžičková et al. [35], the process of biomass combustion produces a diverse range of chemical compounds, which can undergo further oxidation or be directly emitted into the atmosphere. Insights from Bush et al. [72] propose that elevated temperatures (>500 °C) in laboratory settings facilitate the complete breakdown of most herbicides and insecticides. In contrast, lower, smoldering temperatures (<500 °C) may only partially decompose certain pesticides, allowing significant volatilization. Contrary to a sole emphasis on temperature, Růžičková et al. [35] argue that pesticide decomposition is not exclusively determined by temperature. They emphasize the critical role of burning rate, a key factor influencing thermal degradation regardless of the atmospheric conditions.

Focusing on herbicide atrazine, a pivotal compound identified in TSP, PM_10_, and aerosol post-burning, Matuschek et al. [73] conducted a significant thermal analysis study on triazines, including atrazine, metamitron, and metribuzin. Their findings indicated that atrazine degradation in an air atmosphere initiates before its melting point, progressing through four steps, reaching approximately 73% degradation. Interestingly, the observed occurrence of atrazine being found at higher concentrations in the air after burning, despite its lower initial application concentration in raw biomass as compared to carbendazim and chlorpyriphos, raises the need for exploring potential explanations. Discrepancies in the compounds’ volatility, combustion behavior, or transformation processes during biomass burning could be contributing factors. Atrazine may exhibit characteristics that enhance its release into the air, resist degradation during combustion, or undergo transformations resulting in increased concentrations. The complex interplay of various factors, including the compound’s chemical structure, environmental conditions, and interactions with other substances, likely plays a role in this intriguing concentration disparity. Recognizing this complexity, future studies are essential to delve deeper into understanding the mechanisms behind these observations.

### 4.4. Potential Pathways for the Degradation/Transformation of Main Pesticides during Open Burning

Prior to combustion, the rice straw in this study contained a mix of pesticides. Chlorpyriphos was the dominant pesticide at 79.7%, atrazine and its derivatives constituted 15.2%, and 2,4-D made up 3.9%. Carbendazim and diuron were also present in the biomass throughout the experiment. As different pyrolysis conditions can cause diverse chemical compounds to decompose during thermal processes, the transformation of the main pollutants identified in the study is discussed with support from previous research.

In the case of the herbicide atrazine, which stands out as a significant compound detected in TSP, PM_10_, and aerosol samples post-burning, the elucidation of its thermal degradation pathway presents a complex challenge. Only the study by Książczak et al. [74] has provided insights into the thermal decomposition pathway of atrazine, employing thermogravimetric analysis (TG). According to their findings, the TG analysis revealed a three-stage degradation process for atrazine. The initial step involves the removal of alkyl groups, followed by the elimination of the ethyl group to form ethylene. The third step is associated with the removal of chlorine. Similar steps were observed in the degradation of atrazine metabolites, leading to the formation of four decomposition products, namely de-ethyl-deisopropyl-atrazine (DEIA), desethyl-atrazine (DEA), desisopropyl-atrazine (DIA), and hydroxyatrazine. Their results also indicated that an increase in amino groups within the triazine ring corresponds to a higher amount of non-volatile thermal degradation products. The presence of chlorine substituents facilitates the formation of products with low volatility, and hydroxyatrazine undergoes decomposition in a single-stage process. Furthermore, Vikelsøe and Johansen [75] conducted a study evaluating dioxin (PCDD/F) formation during combustion, where six pesticides, including atrazine and diuron, were found to emit the most toxic dioxin congeners (PCDD/F). Specifically, the combustion study identified the release of 2,3,7,8-tetrachlorodibenzo-p-dioxin (TCDD) or 2,3,7,8-tetrachlorodibenzofuran (TCDF).

In the examination of diuron, Gomez et al. [76] conducted a comprehensive study on its thermal decomposition by pyrolysis at temperatures ranging from 400 to 1000 °C in a helium atmosphere. Their findings indicated that dimethylamine was the sole amine detected during diuron pyrolysis. Furthermore, GC-MS analysis revealed the presence of chlorobenzene, 1,2-dichlorobenzene, benzonitrile, trichlorobenzene, aniline, 4-chloroaniline, and 3,4-dichloroaniline. Additionally, the pyrolysis process of diuron resulted in the emission of carbon monoxide (CO), hydrogen cyanide (HCN), and hydrogen chloride (HCl). The initial breakdown involved the cleavage of N-CO bonds, producing isocyanate and amine. Subsequently, these intermediates degraded into substituted ureas and various gaseous products.

Despite numerous proposed degradation pathways for the fungicide carbendazim, the intricate details of its degradation mechanism remain elusive and not fully understood, as highlighted by Liu et al. [77]. A comprehensive study by Senneca et al. [78] stands out as the primary investigation into the thermal decomposition of benomyl and carbendazim in the presence of oxygen. According to their findings, both benomyl and carbendazim undergo multiple stages of decomposition when subjected to heating in an oxidizing atmosphere. Distinctly, benomyl exhibits a first decomposition stage at 120 °C, a characteristic absent in the thermal decomposition of carbendazim. This divergence is likely attributed to differences in molecular structures between the two compounds. Moreover, the degradation pathway of carbendazim may find potential explanation by considering the photolytic degradation pathway, where heat acts as an accelerating factor. Kiss and Virág [79] detailed the photolytic degradation of carbendazim, indicating the initial step involves methyl group loss and the formation of benzimidazole-2-ylcarbamic acid. This product transforms into 2-amino-benzimidazole (2-AB), further converting to stable benzimidazole through deamination. UV irradiation opens the imidazole ring, resulting in 2-methyl-amino-aniline. Contrary to Kiss and Virág [79], Mazellier et al. [80] reported additional products such as 2,4-amino-benzimidazole, benzene, phenol, aniline, and other dimers in their investigations.

In a manner akin to previous pesticides, while there exists substantial information regarding the environmental fate and health impacts of chlorpyriphos [81], there is a noticeable gap in knowledge concerning the effects of fire or heat on chlorpyriphos, as well as its degradation product TCP. Kennedy and Mackie [82] shed light on two pivotal mechanisms in the thermal decomposition of chlorpyriphos. The first pathway involves the formation of TCpyol from chlorpyriphos, with ethoxy group loss in two steps through hydrogen atom transfers, leading to the generation of TCpyol. The second pathway involves oxidative decomposition, initiated by phenolic hydrogen abstraction, resulting in phenoxy radical formation. The radicals combine in a dioxin-like process, leading to consecutive chlorine atom fission and the formation of trans-configured TCDDpy. Weber et al. [83] further indicated that under oxidative conditions the thermal decomposition of chlorpyriphos leads to the rapid attack of TCpyol from CPF by O_2_, O, and OH, resulting in the formation of highly toxic TCDDpy conformers. Undesirable incomplete combustion byproducts such as HCN, HCl, SO_2_, and CO, along with carbon dioxide (CO_2_), are substantial air pollutants resulting from the oxidation of chlorpyriphos under stoichiometric or lean conditions.

It is important to acknowledge that the compounds detected in this study may differ from those reported previously. The complex composition of pesticides presents in commercial formulations, including but not limited to atrazine, diuron, carbendazim, and chlorpyriphos, adds an additional layer of complexity to the analysis. Some compounds may resist thermal decomposition during burning, giving rise to alternative chemical transformations over time. These transformations can involve reactions with various compounds and are susceptible to influence from environmental factors. The specifics of these transformations depend on factors such as the chemical structure of the pesticides, combustion conditions, and the presence of other substances, including oxygen. Consequently, the inherent complexity of this composition plays a substantial role in shaping both the formation and degradation processes of specific compounds detected in TSP, PM_10_, and aerosol. These results align with Chen et al.’s [59] findings, highlighting that the production of toxic byproducts during combustion depends on fire conditions and material response. Incomplete combustion like open burning increases the potential for more toxic species through the fragmentation and rearrangement of original substances. This heightened toxicity is particularly attributed to elements like sulfur, nitrogen, phosphorus, and chlorine in pesticide structures [59].

### 4.5. Presence of PAHs following the Burning of Pesticide-Contaminated Biomass

In the context of PAHs, the incomplete combustion of agricultural residues stands out as a well-documented and substantial source of PAH emissions into the atmosphere [84,85,86]. PAHs, recognized as persistent organic pollutants, pose significant risks to the environment, animals, and public health, particularly in their higher-molecular-weight forms [86,87,88,89]. This study delves into the burning of biomass contaminated with mixed pesticides, revealing a more intricate scenario in both TSP and PM_10_. Remarkably, there were substantial increases in the concentrations of carcinogenic PAHs, collectively constituting approximately 90.1% and 86.9% of all detected PAHs in TSP and PM_10_, respectively. Moreover, the simplified view suggests that incomplete combustion of organic compounds, including pesticides, may generate PAHs through three primary mechanisms: acetylene additions [90], vinylacetylene additions [91], and radical reactions [92]. Reizer et al. [93] proposed “bottom-up” mechanisms to describe the formation of PAHs from smaller molecules. It is important to highlight that in addition to these mechanisms alternative “top-down” processes may take place. These processes involve the fragmentation of amorphous carbon material and can occur in various environments, including both astrophysical settings and combustion processes.

The pathways observed in this study are likely influenced by a combination of factors including biomass properties, pesticide chemical structures, and combustion conditions. Zhang et al. [43] proposed that biomass with high volatile contents can generate abundant phenyl radicals during burning, resulting in significant PM-bound PAH emissions. This observation may be particularly relevant to biomass containing pesticides with volatile properties, suggesting a potential mechanism for PAH release during combustion. Additionally, McGrath et al. [94] illustrated that low-molecular-weight and medium-molecular-weight PAHs are prominently emitted during biomass burning at temperatures equal to or higher than 400 °C, while high-molecular-weight PAHs are more likely to form at even higher temperatures (≥500 °C). Furthermore, the emission of PAHs tends to increase within a specific temperature range, with higher temperatures favoring the synthesis of PAHs from fragments produced by the pyrolysis of biomass during burning [43]. Considering the role of oxygen supply, De Gennaro et al. [95] highlighted its significance in influencing PAH emissions during biomass burning. Their study revealed that biomass burning in a fireplace led to elevated PAH emissions compared to burning in a woodstove.

While certain PAHs may not inherently possess carcinogenic properties, it is crucial to acknowledge that upon release into the atmosphere gas-phase PAHs can undergo transformations, potentially giving rise to more potent carcinogenic and mutagenic forms, such as nitro-PAHs [96]. The observed decrease in concentration within PM_10_ raises intriguing questions about the intricate behavior of PAHs in the atmosphere. This phenomenon may suggest that specific compounds exhibit a preference for associating with larger particles found in TSP. These diverse behaviors point to varied interactions between PAHs and particulate matter, potentially influenced by factors such as particle size, molecular weight, and composition [97]. Given the complex nature of pesticides and PAHs, it is reasonable to anticipate the discovery of new mechanisms in the future, contributing to a deeper and more nuanced understanding of PAH formations and their interactions within environmental matrices.

## 5. Conclusions

The study systematically examined the concentration and diversity of pesticides and PAHs in soil, raw biomass, ash residue, TSP, PM_10_, and aerosol samples before and after the burning of biomass contaminated with mixed pesticides. In untreated soil, propyzamide dominated at 67.6%, while burning led to significant reductions in cyanazine, propyzamide, and triadimefon. Pesticides persisted in raw biomass, with chlorpyriphos dominating at 79.7%, and changes were observed in ash residue post-burning. TSP and PM_10_ showed increased concentrations of various pesticides after burning, with atrazine and chlorpyriphos prominent. Carcinogenic PAHs in TSP and PM_10_ significantly increased after burning. Aerosol analysis revealed a significant post-burning surge in atrazine, accompanied by sebuthylazine, formothion, and propyzamide, albeit at lower levels than TSP and PM_10_. These findings underscore the need for targeted mitigation strategies and comprehensive monitoring initiatives to assess the lasting impacts of open biomass burning on environmental and human well-being. Minimizing the release of persistent pesticides, understanding contaminant fate during burning, and developing approaches to mitigate potential risks associated with post-burning residue are crucial. Advocating for sustainable residue utilization, such as biomass conversion technologies, can minimize environmental impact and promote circular and sustainable use of agricultural residues. Informed, science-based decision-making processes are essential for guiding pathways to enhance food production, ensure food safety, and safeguard the environment. The study only focuses on pesticide application before harvesting, underlining the importance of post-harvest investigations with consideration for withdrawal periods. Future research should explore the transformation of pesticides in straw after withdrawal periods, offering insights into actual rice cultivation conditions. Additionally, there is a need for studies to examine the specific pesticide contamination of biomass, especially straw, when subjected to random and uncontrolled burning conditions. Understanding the thermal decomposition of various chemical compounds at different temperatures, particularly during uncontrolled burning like bonfires, is essential. Recognizing variations in the initial forms of pesticides and their resulting decomposition products in the environment is crucial for a comprehensive assessment of the environmental impact linked to uncontrolled biomass combustion.

## Figures and Tables

**Table 1:** Main composition of the pesticide mixture employed in the investigation.

Chemical Substance	Composition (%)
Chlorpyriphos	90.20
Atrazine	4.64
Diuron	3.62
Carbendazim	1.26
Propazine	0.12
Terbuthylazine	0.10
Simazine	0.05

**Table 2:** Comparing pesticide levels (mg/kg) in soil and straw biomass pre- and post-burning of biomass.

Detected Pesticides	Limit of Detection (LOD)	Soil		Straw Biomass	
		Pre-Burning ^1^ (N = 3)	Post-Burning (N = 3)	Pre-Burning (N = 3)	Post-Burning (N = 3)
**Herbicide**					
Atrazine-desethyl	0.000	ND ^2^	0.084 ± 0.013	ND ^2^	0.158 ± 0.030
Cyanazine	0.000	0.027 ± 0.008	0.003 ± 0.000	0.083 ± 0.007	ND
Simazine	0.145	ND	ND	ND	ND
Atrazine	0.000	ND	2.547 ± 0.139	ND	ND
Propazine	0.000	ND	ND	ND	ND
Sebuthylazine	0.000	ND	ND	ND	ND
Deisopropylatrazine	0.060	ND	ND	ND	ND
Terbuthylazine	0.962	ND	ND	3.484 ± 0.224	5.747 ± 0.152
2,4-D	0.000	0.102 ± 0.001	ND	0.896 ± 0.121	0.473 ± 0.005
Diuron	0.425	ND	ND	ND	ND
Linuron	0.082	ND	ND	ND	ND
Metobromuron	0.000	ND	ND	ND	ND
Ametryn	0.000	ND	ND	ND	ND
**Fungicide**					
Carbendazim	0.774	ND	ND	ND	ND
Triadimefon	0.015	0.110 ± 0.005	0.040 ± 0.010	0.182 ± 0.033	0.024 ± 0.004
Kresoxim methyl	0.000	ND	0.095 ± 0.004	ND	ND
Triadimenol	0.000	ND	ND	ND	0.155 ± 0.028
Prochloraz	0.000	ND	ND	ND	ND
**Insecticide**					
Chlorpyriphos	1.242	ND	52.664 ± 3.410	18.285 ± 1.567	28.303 ± 2.610
Formothion	0.000	0.348 ± 0.029	ND	ND	ND
Propyzamide	0.000	1.221 ± 0.245	0.119 ± 0.005	ND	ND

All values show the mean ± standard deviation. ^1^ Pre-burning refers to raw topsoil and raw straw biomass without mixed pesticides application. ^2^ ND = Not detected.

**Table 3:** Comparing pesticide levels (mg/kg) in total suspended particulate (TSP), particulate matter (PM_10_), and aerosol pre- and post-burning of biomass.

Detected Pesticides	TSP		PM_10_		Aerosol	
	Pre-Burning (N = 3)	Post-Burning (N = 3)	Pre-Burning (N = 3)	Post-Burning (N = 3)	Pre-Burning (N = 3)	Post-Burning (N = 3)
**Herbicide**						
Atrazine-desethyl	0.375 ± 0.019	67.428 ± 2.652	0.438 ± 0.047	23.883 ± 1.231	ND	ND
Cyanazine	0.036 ± 0.004	5.321 ± 0.123	0.038 ± 0.001	4.055 ± 1.041	ND	ND
Simazine	ND ^1^	30.925 ± 1.589	ND	11.910 ± 0.516	ND	ND
Atrazine	18.501 ± 0.574	263.447 ± 11.155	142.291 ± 17.578	426.979 ± 4.468	0.074 ± 0.011	2.915 ± 0.063
Propazine	ND	133.557 ± 0.478	ND	73.593 ± 2.967	ND	ND
Sebuthylazine	0.013 ± 0.019	0.321 ± 0.057	0.032 ± 0.002	0.246 ± 0.024	ND	0.005 ± 0.000
Deisopropylatrazine	0.085 ± 0.07	1.295 ± 0.396	ND	0.157 ± 0.036	ND	ND
Terbuthylazine	ND	107.552 ± 3.059	1.477 ± 0.174	56.272 ± 0.763	ND	ND
2,4-D	ND	0.031 ± 0.009	ND	0.007 ± 0.010	ND	ND
Diuron	3.757 ± 0.712	68.635 ± 1.620	4.306 ± 0.375	40.313 ± 0.558	ND	ND
Linuron	136.963 ± 10.112	15.323 ± 0.023	167.450 ± 3.560	75.631 ± 5.780	ND	ND
Metobromuron	0.125 ± 0.029	59.904 ± 5.498	ND	39.666 ± 8.046	ND	ND
Ametryn	0.478 ± 0.027	29.647 ± 0.582	1.593 ± 0.351	9.378 ± 0.304	ND	ND
**Fungicide**						
Carbendazim	ND	119.288 ± 10.058	ND	24.043 ± 2.765	ND	ND
Triadimefon	1.608 ± 0.200	4.503 ± 1.854	1.524 ± 0.730	3.188 ± 1.119	ND	ND
Kresoxim methyl	0.434 ± 0.014	0.202 ± 0.009	0.414 ± 0.033	0.198 ± 0.022	ND	ND
Triadimenol	34.060 ± 5.497	45.236 ± 1.921	41.239 ± 1.490	75.388 ± 3.032	ND	ND
Prochloraz	41.993 ± 0.816	55.042 ± 0.680	56.289 ± 2.130	60.940 ± 1.109	ND	ND
**Insecticide**						
Chlorpyriphos	ND	347.081 ± 50.402	ND	73.655 ± 4.239	ND	ND
Formothion	24.845 ± 1.656	43.474 ± 4.494	16.868 ± 4.230	64.540 ± 2.970	ND	0.035 ± 0.008
Propyzamide	1.310 ± 0.232	3.777 ± 0.369	1.366 ± 0.092	5.253 ± 0.817	ND	0.032 ± 0.006

All values show the mean ± standard deviation. ^1^ ND = Not detected.

**Table 4:** Polycyclic aromatic hydrocarbon (PAH) levels (ng/m^3^) in total suspended particulate (TSP) and particulate matter (PM_10_) samples before and after application of mixed pesticides.

PAHs	Limit of Detection (LOD)	TSP		PM_10_	
		Pre-Burning (N = 4)	Post-Burning (N = 3)	Pre-Burning (N = 4)	Post-Burning (N = 3)
Nap	1.19	<LOD	<LOD	1.56 ± 0.67	<LOD
Acy	1.17	1.38 ± 0.68	ND	1.81 ± 0.83	ND
Ace	1.02	5.88 ± 3.01	9.41 ± 0.30	8.34 ± 6.09	3.14 ± 0.12
Flu	0.71	1.05 ± 0.52	5.49 ± 0.13	1.00 ± 0.54	0.80 ± 0.03
Phe	1.45	ND ^1^	39.19 ± 0.26	<LOD	6.68 ± 0.12
Ant	0.54	1.13 ± 0.54	ND	0.77 ± 0.36	ND
Fla	1.83	<LOD	14.11 ± 0.55	<LOD	<LOD
Pyr	1.90	ND	1.94 ± 0.13	2.35 ± 1.99	ND
BaA *	1.06	1.67 ± 1.34	180.97 ± 0.83	<LOD	14.88 ± 0.54
Chr *	2.65	<LOD	212.39 ± 0.71	ND	15.65 ± 0.48
BbF *	1.55	<LOD	321.77 ± 0.83	1.59 ± 0.82	75.73 ± 0.37
BaP *	0.58	0.94 ± 0.82	129.63 ± 0.92	0.83 ± 0.41	34.30 ± 0.38
IcdP *	1.10	ND	52.57 ± 0.65	<LOD	13.11 ± 0.41
DahA *	1.84	ND	44.80 ± 0.59	ND	10.31 ± 0.34
BghiP	0.84	ND	33.98 ± 0.63	ND	14.12 ± 0.56

* Carcinogenic PAHs. All values show the mean ± standard deviation. ^1^ ND = Not detected.

## Data Availability

Data will be made available on request.
